# Secret Remedies

**Published:** 1846-12

**Authors:** 


### ARTICLE XV.

### SECRET REMEDIES.

Of all the impositions upon a credulous community, the traffic in nostrums is perhaps the most successful, and carried to the greatest extent. You cannot open a newspaper, or turn a corner, but you are met with an announcement of some *wonderful* elixir of health, whose *undoubted* virtues are blazoned forth by high, sounding recommendations, and hosts of certificates.

It was formerly the case that they pretended to cure all disease, but fashions change in this as in other matters, and we find them now strongly condemning the quackery of this course, (on the principle of the thief crying stop thief,) and confining their application to a few diseases.

In these nostrums the secrecy is the charm which causes community to repose confidence in, and patronize them. Remove this charm, and the speculation in them is at an end. An instance directly in point is fresh in the recollections of the people of the whole West. A few years ago, Sappington's Pills were in almost every store in the country, meeting with the most active demand. Sappington published the recipe, and although a much better and more active preparation than ninety-nine out of a hundred of their class, where are they? almost entirely forgotten.

The legislature of the State of Maine seems to understand this matter, and to conclude that the people will be quite as likely to appreciate the virtues of a remedy, and be much better judges of its value if they know its composition.

They have passed a law providing under heavy penalties, that "no medicine shall be exposed to sale without a label setting concisely the names of all the ingredients or simples of which such medicine is composed, and the proportion of each." We hope, as the good people of the west have been sufficiently gulled by the venders of these, many of them vile compounds, they will put a stop to them by requiring their composition to be given.

But, there is another point in this case to which we wish to refer. It is in the unprofessional conduct of some physicians, in endeavoring to gain reputation with the people by professing to have secret remedies. For the sake of the credit of our profession, we had hoped that no member of it, who claims a higher standing than the empyric, would have condescended to pursue such a course. Of course they place themselves in the same category with the venders of secret remedies, with this difference, that while they acquire a neighborhood notoriety the nostrum vender's name goes abroad through all the land.

We call upon all honorable members of the profession—all who desire to see it maintain a reputation for skill and usefulness—who desire to see a clear line of distinction drawn between the quack and the physician—the ignorant pretender and the man of science, to set their faces against all such disreputable practices and gross deceptions. E.

